# IVF and Thermal Manipulation at the First Cleavage Stage Alter Offspring Circadian Phenotype, Sleep, and Brain Epigenetics

**DOI:** 10.3390/ijms262110360

**Published:** 2025-10-24

**Authors:** Daniil Zuev, Aliya Stanova, Galina Kontsevaya, Alexander Romashchenko, Nikita Khotskin, Marina Sharapova, Mikhail Moshkin, Ludmila Gerlinskaya, Yuri Moshkin

**Affiliations:** 1 Federal Research Center Institute of Cytology and Genetics, Siberian Branch of Russian Academy of Science (RAS), Novosibirsk 630090, Russia; zuevdaniil.zuevdaniil@gmail.com (D.Z.); khotskin@bionet.nsc.ru (N.K.); mmp@bionet.nsc.ru (M.M.); 2 Department of Vertebrate Zoology and Ecology, Institute of Biology, Ecology, Soil Science, Agriculture and Forestry, Tomsk State University, Tomsk 634050, Russia; 3 Gene Learning Association, 1205 Geneva, Switzerland

**Keywords:** epigenetics, IVF incubation temperature, IVF-conceived offspring, energy balance, circadian rhythms, sleep architecture, brain histone modifications

## Abstract

In vitro fertilization (IVF) exposes embryos to environmental stressors that can disrupt early development and confer long-term health risks, though the mechanisms remain poorly understood. Here, we tested the hypothesis that reducing incubation temperature during the first zygotic cleavage would promote long-term developmental stability in IVF-conceived offspring. Using a mouse model, we compared the long-term effects of standard (37 °C) versus reduced (35 °C) IVF culture temperature on energy balance, circadian rhythms, sleep architecture, and brain histone modifications. Although offspring from both IVF groups exhibited increased body mass without notable effects on glucose metabolism, significant disruptions in circadian rhythms and sleep–wake patterns were detected. The 37 °C group exhibited altered amplitudes in oxygen consumption rhythms and respiratory exchange ratios, as well as pronounced alterations in sleep–wake patterns, including reduced sleep duration and increased nighttime activity. The 35 °C group displayed intermediate phenotypes, substantiating the importance of optimizing embryo incubation parameters. These metabolic and behavioral changes were paralleled by altered histone modifications in the cerebral cortex of IVF offspring, suggesting an epigenetic basis for circadian misalignment. Our results identify disrupted circadian rhythm and sleep architecture as a novel mechanism contributing to metabolic dysfunction in IVF-conceived offspring. The partial mitigation of these effects through reduced culture temperature underscores the importance of optimizing IVF protocols to minimize long-term epigenetic and metabolic risks.

## 1. Introduction

The global incidence of metabolic diseases has risen markedly in recent decades, reaching a pandemic scale; the WHO reports that over a third of adults are obese across many Western nations. For children conceived via assisted reproductive technologies (ART), the risk of such metabolic disorders is elevated even further [1]. This risk is associated with alterations during prenatal development from ART procedures, which may program metabolic dysfunction later in life [2]. Although evidence confirms an increased risk of both short- and long-term metabolic complications following ART, a consensus on the precise causes is lacking [1,3,4,5].

The origin of metabolic complications in offspring conceived via assisted reproductive technology (ART) is debated [6,7]. While some attribute these effects to parental health factors, mouse studies demonstrate that the IVF (in vitro fertilization) procedure itself is a direct cause. For instance, research indicates that IVF-conceived mice exhibit a higher predisposition to metabolic syndrome, disrupted carbohydrate homeostasis, obesity, and diabetes mellitus compared to those conceived naturally [4,8,9,10]. Although studies on people conceived via ART are confounded by factors like parental health and young cohort age, emerging evidence also suggests a link between ART and elevated risks of metabolic alterations, such as increases in fasting glucose, insulin resistance, and accumulation of peripheral body fat [11,12,13].

Visceral fat accumulation elevates the risk of developing metabolic syndrome [14,15], primarily due to an imbalance between energy substrate consumption and energy expenditure. Among other factors, disruption of circadian rhythms may cause such an imbalance [16,17,18,19]. Supporting this idea, it has been shown that the diurnal rhythm of food consumption was altered in the IVF-conceived male CD1 mice, and that these mice had increased body mass compared to the naturally conceived ones, despite equivalent food intake [4].

The circadian clock is based on transcriptional-translational feedback [20,21], and it coordinates vital biological pathways, including the cell cycle, DNA replication/repair, and energy metabolism [22,23,24,25]. Circadian and metabolic gene regulation is tightly controlled by DNA and histone epigenetic marks [20,26,27,28]. Furthermore, a bidirectional relationship exists between the circadian clock and metabolic regulation. The core clock machinery directly regulates the expression of metabolic genes, while metabolic signals provide feedback to the circadian clock. Thus, disruption of the circadian rhythm leads to conditions such as obesity and metabolic syndrome, while metabolic dysregulation has an impact on the circadian system [29,30,31]. Although evidence for a direct connection between ART and circadian disruption is limited [4], it can be suggested that IVF-mediated epigenetic alterations may lead to circadian misalignment, which in turn may contribute to the metabolic disruptions observed in IVF-conceived offspring. This notion is supported by the research showing that the IVF procedure in mice results in increased cell-to-cell variations in DNA methylation (5mC) in early-stage blastomeres, indicating a destabilized epigenetic landscape [32]. Likewise, in humans, ART induces epigenetic alterations, particularly at imprinted genes, that persist from birth into later life. Consequently, this increases susceptibility to a range of imprinting disorders, including metabolic and cardiovascular diseases, as well as specific conditions such as Angelman Syndrome, which is linked to a disrupted circadian rhythm [3,33,34,35]. Although the autonomous circadian clock oscillations are activated only shortly before gastrulation [36], the expression of Clock and Per1 genes starts immediately after the zygotic genome activation (ZGA), at two- and four-cell stages for mice and humans, respectively [36,37]. This makes the period of initial cleavage and ZGA a critically sensitive window for establishing robust epigenetic regulation of circadian networks.

Temperature plays a crucial role in stabilizing epigenetic and transcriptional fluctuations, and it helps synchronize the clock genes through the direct modulation of chemical reaction rates [38,39]. Notably, in vivo fertilization and first zygotic cleavages occur 1–3 °C below core body temperature, suggesting that standard IVF culture conditions at 37 °C may represent a non-physiological thermal stress [40]. In mouse IVF embryos, intra-blastomere variability in 5mC genomic methylation, a sign of epigenetic fluctuation, is minimized at lower incubation temperatures by the eight-cell blastomere stage compared to the normal incubation conditions at 37 °C [32]. Likewise, lower incubation temperatures for IVF embryos reduce transcriptional fluctuations, as indicated by decreased gene expression fluctuating asymmetry in the resulting offspring [41,42]. The lowered incubation temperatures of IVF embryos also increase newborns’ survival, despite increased embryo losses [32]. These effects are consistent with broader mechanisms of temperature’s influence on chromatin, including the stabilization of MNase-sensitive nucleosomes [39] and chromatin condensation driven by a temperature-induced influx of calcium [38,43]. This temperature-sensitive chromatin remodeling provides a potential mechanism by which thermal manipulation during early zygotic cleavage could alter developmental epigenetic trajectory, potentially influencing the regulation of circadian rhythm and subsequently metabolic processes throughout life.

Here, we sought to investigate the impact of IVF and incubation temperature during initial cleavage on the circadian regulation of metabolism, behavior (including feeding, activity, and sleep), and brain epigenetics in the CD1 male mouse offspring. We hypothesized that IVF would cause lasting epigenetic alterations in offspring, which, in turn, could disrupt their circadian rhythm and metabolic health. We further proposed that lowering the incubation temperature to 35 °C during the first cleavage division would stabilize the embryo’s epigenome, thereby alleviating these adverse phenotypes. We have demonstrated that IVF conception leads to increased body mass associated with a disruption of the sleep–wake cycle, which subsequently alters other metabolism-related parameters such as spontaneous locomotor activity, food intake, and respiratory exchange ratio. Concomitantly, we observed alterations in cortical active and repressive histone methylation marks (H3K27me3, H3K36me3, H3K9me2). The phenotypic and epigenetic deviations from naturally conceived controls were mitigated by incubating embryos at 35 °C rather than 37 °C during the initial zygote cleavage stage.

## 2. Results

### 2.1. IVF Offspring Exhibit Increased Body Mass Without Glucose Intolerance

To assess the impact of IVF and embryo incubation temperature on offspring, we fertilized CD1 mouse oocytes and incubated them at either 35 °C or 37 °C for 24 h. We used the outbred CD1 mouse strain to minimize genetic bias and to extend our previous investigations into the effects of IVF and incubation temperature on developmental, epigenetic, and metabolic outcomes [32,41]. The resulting two-cell embryos were transferred to pseudo-pregnant CD1 female mice. All subsequent experiments were conducted on the male IVF offspring, using naturally conceived male CD1 offspring as controls.

**Figure 1:**
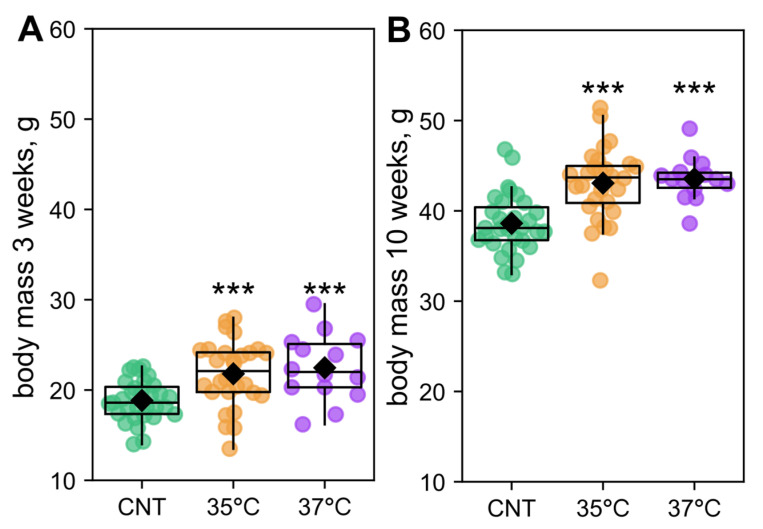
Body mass of male offspring at (**A**) 3 weeks and (**B**) 10 weeks of age. *** *p* < 0.001 ANCOVA Fisher LSD post hoc. The number of male offspring was 31 in the control (CNT) group, 28 in the 35 °C IVF group and 14 in the 37 °C IVF group.

Male offspring mass was assessed at 3 and 10 weeks of age (Figure 1). We compared the groups using ANCOVA, treating the experimental group (control—CNT, and two IVF groups—35 °C, 37 °C) as a categorical factor and the number of littermates as a confounding variable. At 3 weeks, the effect of the group was not significant (F_2,69_ = 0.50, *p* = 0.61), whereas the number of littermates had a highly significant effect (F_1,69_ = 21.6, *p* < 0.001). However, Fisher’s LSD (least significant difference) post hoc test revealed that both the 35 °C and 37 °C groups were significantly heavier than controls (Figure 1A). By 10 weeks, the effect of littermate number was no longer significant (F_1,69_ = 2.82, *p* = 0.098), but the group effect became significant (F_2,69_ = 6.05, *p* < 0.01). Post hoc analysis confirmed that both experimental groups remained significantly heavier than controls (Figure 1B).

**Figure 2:**
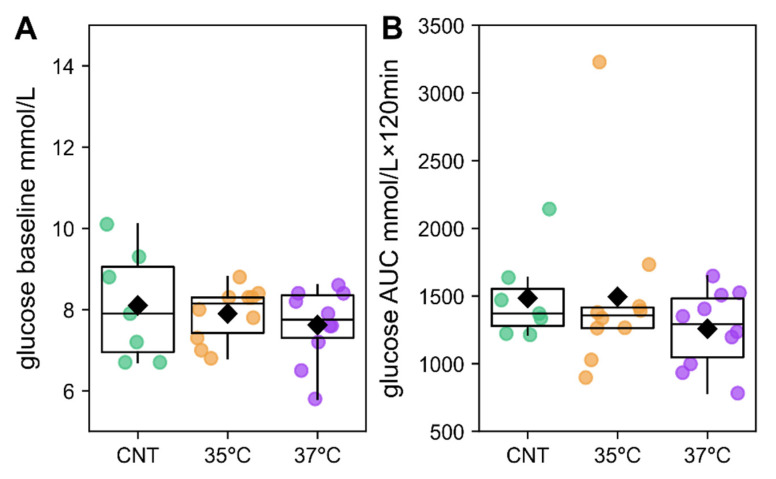
Glucose baseline level (**A**,**B**) area under the curve (AUC) in the glucose tolerance test. AUC was calculated for the first 120 min after glucose administration using the trapezoidal rule. No statistically significant differences were revealed (Mann–Whitney U-test). The number of animals was 7 in the control (CNT) group and 10 in the 35 °C and 37 °C IVF groups.

For subsequent studies, we randomly selected one to two males from each litter. While their initial weight at 3 weeks did not differ significantly between groups, by 10 weeks, the animals from both the 35 °C and 37 °C groups were significantly heavier than the controls (Figure S1). Glucose tolerance was assessed in this selected 10-week-old offspring cohort. Contrary to expectations based on the increased mass of male IVF offspring, these groups displayed no significant differences in baseline glucose levels or glucose tolerance compared to the control (Figure 2).

### 2.2. Metabolic and Behavioral Phenotyping of IVF-Conceived Offspring

**Figure 3:**
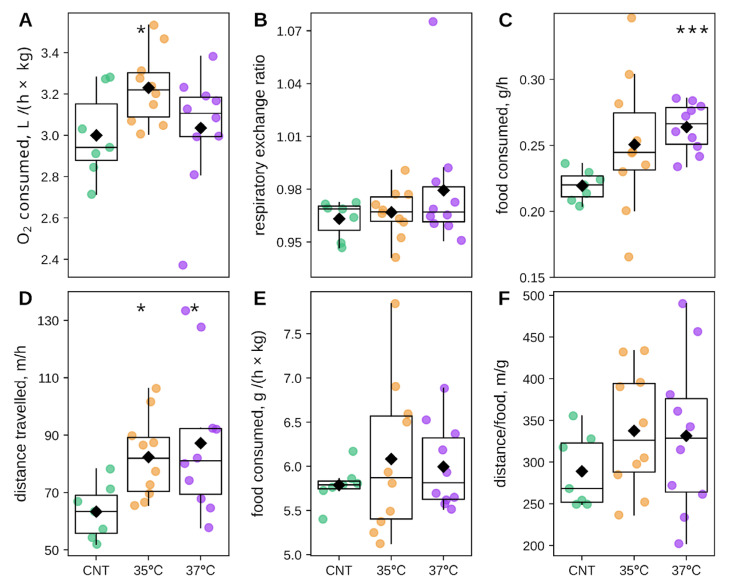
Average metabolic and behavioral metrics. (**A**) Oxygen consumption (liters per hour per kilogram of body weight, L/h/kg), (**B**) Respiratory exchange ratio (RER), (**C**) Food intake (grams per hour, g/h), (**D**) Distance traveled (meters per hour, m/h), (**E**) Mass-specific food intake (g/h per kilogram of body, g/h/kg), (**F**) Food-distance ratio (m/g). * *p* < 0.05, *** *p* < 0.001 Mann-Whitey U-test IVF groups vs. control. The number of animals was 7 in the control (CNT) group and 10 in the 35 °C and 37 °C IVF groups.

To further characterize the offspring, we analyzed averaged metabolic and behavioral time-series data collected using a Phenomaster system. We continuously monitored 10-week-old animals for 4 days (96 h), measuring oxygen consumption, respiratory exchange ratio (RER), food intake, and locomotor activity. The data were analyzed by first calculating the average of the time-series parameters for each individual and then comparing these averages across the different groups (Figure 3).

The average oxygen consumption was significantly elevated in the 35 °C IVF group compared to controls; in contrast, the 37 °C IVF group did not differ significantly from the control (Figure 3A). The average RER did not differ significantly between groups (Figure 3B). Food consumption was significantly greater in the 37 °C IVF group than in controls, while the 35 °C IVF group food intake was intermediate to the 37 °C and control groups but not statistically different from either (Figure 3C). Locomotor activity was significantly higher in both the 35 °C and 37 °C IVF groups compared to the control group (Figure 3D). Food consumption normalized to body weight did not differ between groups (Figure 3E), nor did the distance traveled per gram of food consumed (Figure 3F). These results rule out the possibility that the more active IVF mice compensated for their energy expenditure by disproportionately increasing their food consumption, which could have explained their increased mass gain. In summary, the observed mass gain in both cohorts of IVF-conceived male mice was not attributable to averaged metabolic or behavioral parameters.

### 2.3. Impact of IVF and Incubation Temperature at the First Cleavage on Offspring Circadian Phenotype

**Figure 4:**
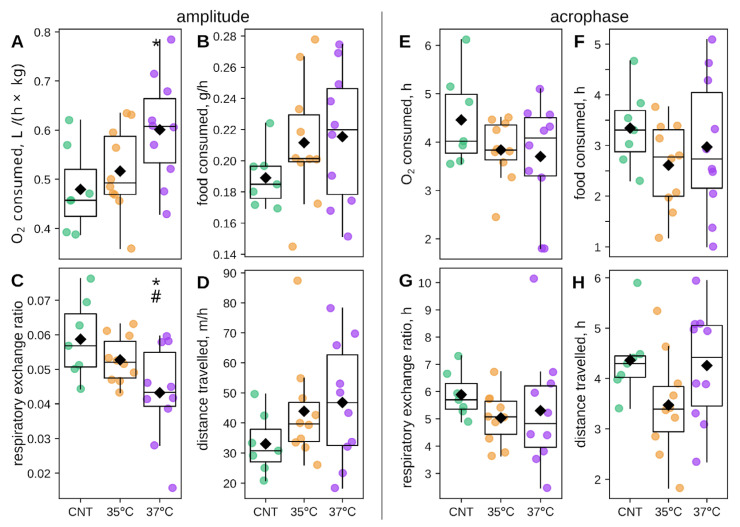
Cosinor analysis of circadian metabolic and behavioral parameters. Amplitude of (**A**) oxygen consumption (L/h/kg), (**B**) food intake (g/h), (**C**) respiratory exchange ratio (RER), and (**D**) distance traveled (m/h). Acrophase (time of daily peak, in hours) for (**E**) oxygen consumption, (**F**) food intake, (**G**) RER, and (**H**) distance traveled. Significance vs. control: * *p* < 0.05 and # *p* < 0.05 for 35 °C vs. 37 °C groups (Mann–Whitney U-test). The number of animals was 7 in the control (CNT) group and 10 in the 35 °C and 37 °C IVF groups.

We next assessed whether the circadian rhythm, specifically the amplitude and phase of oscillations, in metabolic and behavioral parameters differed between IVF and control groups. This was achieved by fitting a cosinor regression model to individual animal time series (Figure S2). The mesor values for each Phenomaster metabolic and behavioral parameters were completely analogous to the differences observed in the time series averages (Figure S3). Interestingly, we found group-specific differences in circadian amplitude: oxygen consumption was increased in the 37 °C IVF group (Figure 4A), whereas RER was decreased in the 37 °C IVF group compared to controls. For the 37 °C IVF group, RER circadian amplitude was also lower compared to the 35 °C IVF group (Figure 4C). Amplitudes for the 35 °C IVF group were intermediate and not significantly different from controls for oxygen consumption and RER. No significant amplitude differences were observed for locomotor activity (distance traveled) or feeding (Figure 4B,D). Acrophase did not differ between groups for any parameter (Figure 4E–H). These amplitude changes in key metabolic parameters indicate distinct circadian metabolic phenotypes.

To determine the temporal distribution of the observed circadian metabolic and behavioral parameters, we averaged time series across experimental days to generate representative 24 h profiles for each animal (Figure S4). Analysis of diurnal profiles revealed distinct patterns for each group. The elevated average oxygen consumption in the 35 °C IVF group (Figure 3A) resulted from a consistent, slight elevation across the entire day, becoming significant at specific time points (Figure S4A). Conversely, the significantly larger amplitude in the 37 °C IVF group (Figure 4A) was not driven by differences at any specific time point (Figure S4A) but rather through a cumulative effect. RER profiles exhibited a distinct pattern (Figure S3B). The 37 °C IVF group showed a pronounced elevation during the latter half of the light phase compared to controls, which explains its reduced amplitude (Figure 4C). The 35 °C IVF group profile was intermediate between those of the 37 °C and control groups. Food consumption was elevated in the 35 °C and 37 °C IVF groups at several time points during the late light phase (Figure S4C). Locomotor activity was significantly higher in both IVF groups at numerous points across the entire 24 h cycle (Figure S4D), a pattern consistent with their elevated daily averages (Figure 3D). In summary, IVF-induced differences in RER and feeding were predominantly confined to the light phase, while alterations in oxygen consumption and locomotion manifested as sustained, circadian-wide increases.

**Figure 5:**
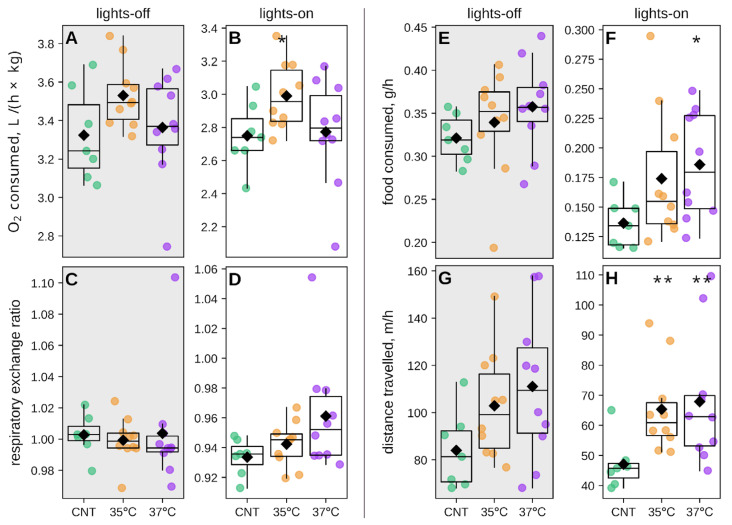
Average metabolic and behavioral parameters during light (lights-on) and dark (lights-off) phases. (**A**,**B**) oxygen consumption (L/h/kg), (**C**,**D**) respiratory exchange ratio (RER), (**E**,**F**) food intake (g/h), (**G**,**H**) distance traveled (m/h). Significance vs. control: * *p* < 0.05, ** *p* < 0.01, (Mann–Whitney U-test). The number of animals was 7 in the control (CNT) group and 10 in the 35 °C and 37 °C IVF groups.

We next calculated the average values for each parameter during the light and dark phases separately (Figure 5). The 35 °C IVF group exhibited significantly elevated oxygen consumption during the dark phase, with a non-significant increase in the light phase (Figure 5A,B). Despite significant differences at specific time points of the light phase (Figure S4B), RER showed no significant differences between groups when averaged across light and dark phases (Figure 5C,D). The 37 °C IVF group consumed significantly more food during the light phase, and both IVF groups showed significantly greater locomotor activity during the light phase compared to controls (Figure 5E–H). Together, these findings demonstrate that IVF-conceived offspring exhibited altered circadian rhythms, with differences predominantly occurring during the diurnal rest (lights-on) phase.

### 2.4. The Effects of IVF and First-Cleavage Incubation Temperature on Offspring Sleep

**Figure 6:**
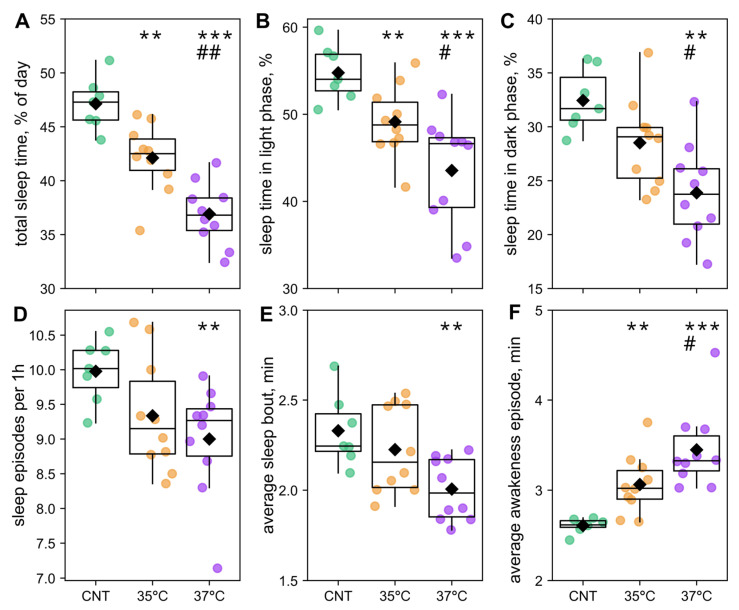
Sleep analysis. The percentage of total sleep time per 24 h circadian cycle (**A**), light phase (**B**), and dark phase (**C**). Sleep episodes per hour (**D**), average time of sleep bout (**E**), and awakeness episode (**F**). ** *p* < 0.01, *** *p* < 0.001 IVF groups vs. control; # *p* < 0.05, ## *p* < 0.01 37 °C vs. 35 °C IVF group (Mann-Whitey U-test). The number of animals was 7 in the control (CNT) group and 10 in the 35 °C and 37 °C IVF groups.

To assess sleep, we analyzed periods of inactivity logged by the Phenomaster system, which can detect short sleep bouts of 2–5 min [44]. Sleep analysis revealed significant, phase-specific alterations in both IVF groups. The percentage of total sleep time was reduced in the 35 °C IVF group and further diminished in the 37 °C IVF group compared to controls (Figure 6A). A similar progressive decrease in the percentage of total sleep occurred during the light (rest) phase across the control, 35 °C, and 37 °C IVF groups. (Figure 6B). During the dark (active) phase, sleep was significantly reduced only in the 37 °C IVF group relative to both controls and the 35 °C IVF group (Figure 6C). Furthermore, sleep architecture was disrupted. The number of sleep episodes per hour and the average sleep bout length were lower in the 37 °C IVF group than in the 35 °C IVF group and controls (Figure 6D,E).

Collectively, the sleep and circadian phenotyping data suggest that IVF conception increases nervous system excitability, leading to reduced sleep, hyperlocomotion, and consequently elevated food intake. These effects are most pronounced during the light (rest) phase and likely contribute to the observed increase in offspring mass. Notably, the intermediary phenotype of the 35 °C IVF group indicates that lowering the first-cleavage embryo incubation temperature mitigates the impact of IVF on circadian phenotype and sleep outcomes.

### 2.5. IVF Mice Have Altered Histone Modification

**Figure 7:**
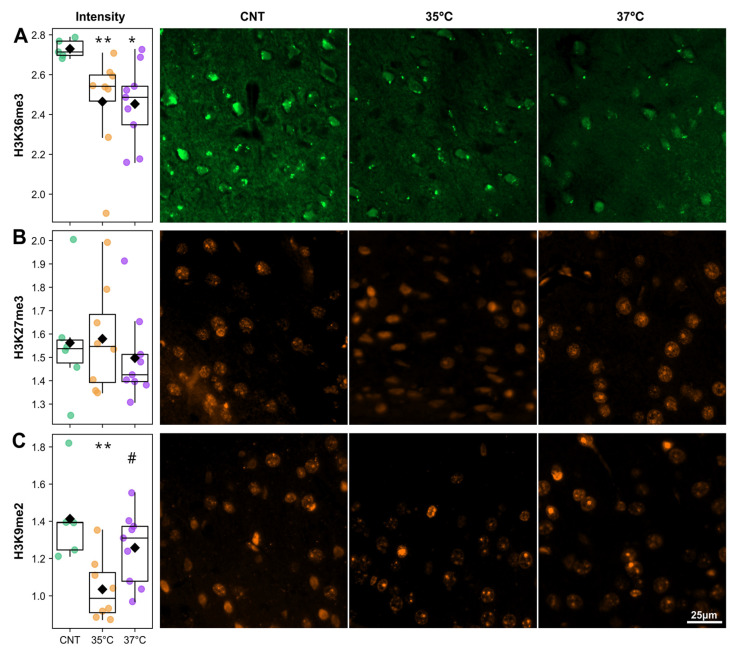
Quantification of histone modification foci in the somatosensory cortex, along with representative histological images for each group. (**A**) H3K36me3, (**B**) H3K27me3, and (**C**) H3K9me2. Histone modification was measured as the mean fluorescence normalized by DAPI signal. * *p* < 0.05, ** *p* < 0.01, IVF groups vs. control; # *p* < 0.05 37 °C vs. 35 °C IVF group (Mann-Whitey U-test).

Although the mechanisms conferring IVF-associated phenotypes are poorly understood, epigenetic instability during zygotic reprogramming, such as the destabilization of 5mC methylation [32], suggests a potential pathway. We therefore evaluated the effect of IVF and incubation temperature on the epigenetic landscape in the somatosensory cortex of 27–28-week-old offspring from the control and IVF (35 °C, 37 °C) groups, focusing on key histone modifications: the active mark H3K36me3 and the repressive marks H3K27me3 and H3K9me2. Immunohistochemical analysis revealed significant, mark-specific alterations in histone modifications, quantified as the mean fluorescence intensity divided by the mean DAPI intensity for each nuclear area. Nuclear areas were automatically segmented from the DAPI channel (Figure S5). The intensity of H3K36me3 fluorescent signal from nuclei areas was significantly decreased in the 35 °C and 37 °C IVF groups relative to controls (Figure 7A). The H3K27me3 positive foci areas were not significantly different between controls (Figure 7B). H3K9me2 staining intensity was significantly lower in the 35 °C IVF group than in both the control and 37 °C IVF groups (Figure 7B). We conclude that IVF and incubation temperature have a lasting impact on the offspring’s cortical epigenetic landscape.

## 3. Discussion

The IVF procedure subjects embryos to environmental stress due to in vitro culture conditions and the elimination of endogenous signaling factors present in the seminal fluid and oviduct. These stressors can disrupt early zygotic development, with potential long-term consequences for implantation, fetal growth, and postnatal health [45,46,47]. For instance, even short-term fluctuations in culture media pH and incubation temperature can significantly impact embryo viability during ART and may have long-term effects on fetal development [48]. Furthermore, the impact of incubation temperature on IVF-conceived offspring is often overlooked, despite physiological evidence that in vivo fertilization and initial zygotic cleavage occur 1–3 °C below core body temperature, i.e., below the standard embryo incubation temperature of 37 °C [40,49].

Although the nature of how long-term effects from embryo culture conditions are conferred to offspring remains largely unknown, it is likely to involve epigenetic modifications [50,51,52]. Indeed, pH fluctuations in embryo culture can drive global changes in histone acetylation [53], while temperature shifts alter chromatin condensation [38,39]. Notably, spontaneous intra-blastomere variations in genomic 5mC DNA methylation and gene expression variability are reduced by the eight-cell stage in mouse embryos cultured at 35 °C compared to 37 °C, reaching levels comparable to naturally conceived embryos [32]. We therefore hypothesized that lowering the incubation temperature during the first zygotic cleavage would confer long-term developmental stability, thereby mitigating adverse outcomes associated with IVF.

In this study, we compared the long-term effects of two IVF culture temperatures (35 °C vs. standard 37 °C) on energy balance, circadian rhythms, and cerebral cortex histone modifications in outbred CD1 mouse offspring, using naturally conceived offspring as a control.

Body mass, an integral indicator of metabolic activity, is elevated in IVF-conceived children [54,55,56]. Consistent with this, offspring in both IVF groups had increased body mass compared to controls. At 3 weeks, this increase was attributable to smaller litter sizes, whereas by 10 weeks, the effect was directly caused by IVF itself. However, incubation temperature had no significant effect on body mass (Figure 1). Contrary to the findings that IVF may impair glucose tolerance in male mouse offspring [57], baseline glucose and glucose tolerance were comparable to controls in both studied IVF groups (Figure 2). Consequently, we investigated the mechanisms by which IVF and its culture temperature influence offspring energy metabolism in greater depth.

Besides glucose metabolism, body mass is governed by energy intake/expenditure balance, e.g., a positive energy balance, whether from increased food intake or reduced energy expenditure, promotes increased body mass [58,59]. Thus, we tracked O_2_ consumption, CO_2_ production, food intake, and spontaneous locomotor activity in 10–11 = week-old IVF and control offspring (Figure 3). Food consumption was significantly increased in the 37 °C IVF group and non-significantly in the 35 °C IVF group compared to controls. Although locomotor activity (distance traveled) was higher in both IVF groups, the energy balance (distance traveled to food consumption ratio) did not differ significantly from controls. This prompted an investigation into the diurnal partitioning of energy intake and expenditure to assess potential links to circadian rhythm disruption.

A misalignment between the circadian timing of energy consumption and expenditure represents a significant risk factor for the development of obesity, type 2 diabetes, and cardiovascular disease [60,61]. Cosinor analysis revealed significant circadian metabolic alterations in the IVF groups (Figure 4). The 37 °C IVF group exhibited a significantly increased amplitude of O_2_ consumption and a decreased RER over the 24 h cycle compared to controls. The 35 °C IVF group showed intermediate, but non-significant, changes in the amplitude of these two parameters. During the rest (lights-on) phase, O_2_ consumption was significantly elevated in the 35 °C IVF group, though RER was unchanged. The 37 °C IVF group showed O_2_ consumption comparable to controls in both phases, with a non-significant trend toward increased RER during the rest phase. These results indicate distinct alterations in circadian energy metabolism patterns in IVF-conceived offspring.

Interestingly, we observed a concomitant increase in spontaneous locomotor activity and food consumption during the rest phase (lights-on) in the IVF offspring, which was most pronounced in the 37 °C IVF group (Figure 5). These behavioral alterations indicate a disruption in circadian sleep–wake patterns. Indeed, analysis of sleep patterns revealed that, compared to controls, the 37 °C IVF group had significantly reduced sleep time (total and during the dark and light phases) and shorter sleep but longer awakeness episode duration. The 35 °C group exhibited intermediate alterations in the sleep–wake phenotype (Figure 6). Therefore, the observed disruptions in circadian energy metabolism and sleep–wake patterns may be key mechanisms driving the accumulation of mass in IVF offspring, with potential implications for their long-term metabolic health. Interestingly, although our findings in mice do not necessarily directly translate to humans, it is notable that IVF-conceived children have a significantly higher cumulative incidence of obstructive sleep apnea [62].

Finally, the observed alterations in circadian rhythm and sleep–wake patterns could be underpinned by epigenetic modifications conferred to offspring. Indeed, chromatin and histone modification dynamics play a crucial role in regulating core circadian clock genes [26,28,63,64]. Consequently, analysis of histone modifications in the somatosensory cortex of IVF offspring supported this hypothesis (Figure 7). The cerebral somatosensory cortex runs an autonomous circadian clock and exhibits diurnal rhythmicity in the activity of excitatory and inhibitory synapses, and it is also implicated in resynchronization of the master clock [65,66,67]. The gross epigenetic alterations in IVF-conceived offspring observed in this study are likely to disrupt the expression of clock genes, which rely on the tight orchestration of epigenetic marks to maintain synchronized oscillations. Clock genes are activated by H3K36me3 and repressed by H3K9me2 and H3K27me3 [63,68,69,70]. A reduction in the active H3K36me3 mark in both IVF groups could lead to a general downregulation of clock genes. However, in the 35 °C IVF group, this could be compensated for by a concurrent global decrease in the repressive H3K9me2 mark (Figure 7). While the direct effects on circadian gene expression require further validation, our findings indicate that IVF causes lasting epigenetic alterations in the offspring’s cerebral somatosensory cortex. These alterations offer a plausible explanation for the dysregulation of circadian rhythms and consequent wide-ranging effects on metabolic health.

Collectively, our results demonstrate that dysregulated sleep–wake Ok patterns and circadian energy metabolism constitute a novel pathway contributing to elevated body mass in IVF offspring. To the best of our knowledge, this is the first study to underscore the IVF-mediated impact on circadian rhythms in offspring, which warrants deeper analysis of circadian alterations in the IVF-conceived population and the implications of ART protocols in driving circadian misalignment. Crucially, the partial amelioration of these phenotypes through reduced incubation temperature underscores that modifying embryo culture conditions in accordance with natural temperature gradients experienced by the zygote during the first cleavages is a promising strategy to attenuate the long-term adverse effects of IVF on offspring. Therefore, elucidating the precise molecular cascade, from temperature-sensitive chromatin remodeling in the zygote to the dysregulation of the central circadian clock in offspring, deserves future research for developing optimal ART/IVF strategies to reduce metabolic and other health risks in ART/IVF-conceived offspring.

## 4. Materials and Methods

### 4.1. Animals

The study was performed on outbred CD1 mice maintained under specific pathogen-free (SPF) conditions. Animals were housed in individually ventilated cages (OptiMice, Centennial, CO, USA) under controlled conditions: a 14:10 h light-dark cycle, 22–24 °C, and 40–50% humidity. Males were housed singly, and females were housed in groups of five, with autoclaved feed and water provided ad libitum. All animal procedures were conducted in compliance with the Standard Operating Procedures (SOP) of the specific pathogen-free (SPF) facility and the Animal Care and Use Committee of the Federal Research Center Institute of Cytology and Genetics (ICG) operating under regulatory guidelines of the Federal Health Ministry (2010/708n/RF) and NRC. The study protocols received approval from the Bioethics Commission of ICG SB RAS (approval No. 20, 3 November 2022).

### 4.2. In Vitro Fertilization, Embryo Transfer, and Offspring Weaning

Virgin CD1 females were superovulated via intraperitoneal (i.p.) injection of 5 IU pregnant mare serum gonadotropin (Folligon, MSD Animal Health, Boxmeer, Netherlands), followed by 5 IU human chorionic gonadotropin (hCG; Horulon, MSD Animal Health, Boxmeer, Netherlands) 48 h later. Cumulus-oocyte complexes (COCs) were collected from the oviductal ampulla 15–17 h post-hCG injection and placed in a 200 μL droplet of human tubal fluid (HTF) medium. For IVF, sperm were collected from the caudal epididymis and placed in a 100 μL HTF drop under mineral oil (Sigma–Aldrich, St. Louis, MO, USA) and pre-incubated for one hour at 37 °C and 5% CO_2_. Then, 3–5 μL of this sperm suspension was added to the fertilization drop containing COCs and incubated for 3–4 h. Fertilized oocytes were washed with four drops of HTF and cultured in 80 μL HTF droplets under mineral oil at 35 °C or 37 °C in 5% CO_2_ for 24 h to the two-cell stage.

Following in vitro culture at 35 °C or 37 °C, two-cell embryos were transferred into pseudopregnant CD1 recipient females under AERRANE anesthesia (Baxter Healthcare Corp., Deerfield, IL, USA). The recipient females were produced by mating 23 CD1 females (10–14 weeks old) with 10 vasectomized males. In total, 199 embryos from the 35 °C IVF group were transferred to 13 females, and 151 embryos from the 37 °C IVF group were transferred to 10 females. For the control, 8 CD1 females (10–14 weeks old) were mated with CD1 males.

After the delivery, the number of newborns and subsequently weaned offspring was counted. In total, 56 (31 males and 25 females) offspring were born and weaned in the control group; 49 (28 males and 21 females) offspring were born and weaned in the 35 °C IVF group; 25 (14 males and 11 females) offspring were born and 23 (14 males and 9 females) were weaned in the 37 °C IVF group.

For further analysis, 1–2 male offspring from each litter were included in the experimental groups, resulting in the following group sizes:

1. control (CNT)—(n = 7),
2. 35 °C IVF group—(n = 10),
3. 37 °C IVF group—(n = 10).

Male offspring were weighed at the ages of 3 and 10 weeks.

### 4.3. Glucose Tolerance

A glucose tolerance test (GTT) was performed on 10–11-week-old male mice. After a 16 h fast, mice were i.p. injected with a 20% glucose solution (PanEco, Moscow, Russia) at a dose of 10 μL per gram body weight. Blood was collected from the tail vein immediately before the injection and at 15, 30, 60, and 120 min after the injection. Blood glucose levels were measured using a Contour TS glucose meter (Bayer, Basel, Switzerland). The area under the curve (AUC) was calculated relative to baseline using the trapezoid rule as an integrated index of glucose tolerance.

### 4.4. Circadian Phenotyping

Circadian phenotyping was conducted on 10-week-old male offspring using a Phenomaster system (TSE, Berlin, Germany). Each animal was housed in an individual Phenomaster cage. Following a 2-day habituation period, data were recorded for 3 days. Measured parameters included spontaneous locomotor activity (distance traveled), food consumption, oxygen consumption (VO_2_), carbon dioxide production (VCO_2_), and sleep episodes. The respiratory exchange ratio (RER) was calculated as the ratio of VCO_2_ to VO_2_. All recordings commenced at the onset of the dark phase (lights-off), designated as Zeitgeber Time 0 (ZT0), and continued for approximately 100 h.

Phenomaster time series data were processed and analyzed as follows. The raw data were first aggregated into 30 min bins to create time series with uniform sampling frequency and the same length. From these, overall and light-phase-specific (lights-on/lights-off) averages were calculated. Circadian rhythms were assessed by fitting cosinor regression models to estimate acrophase and amplitude. A classical cosinor model was applied to oxygen consumption and respiratory exchange data (Figure S2A), whereas distance and feed consumption were log-transformed before fitting (Figure S2B). The cosinor model is parameterized by three variables: the mesor (constant term), the amplitude of oscillation, and the acrophase. The acrophase is reported in hours for clarity. Finally, a periodic average was generated to visualize the trend in average daily activity (Figure S4). All analyses were performed using Python version 3.13 (library versions: numpy 2.2, pandas 2.3).

### 4.5. Immunofluorescent Analysis of Histone Modifications in the Somatosensory Cortex

Male offspring (27–28 weeks of age) from the above-described groups were deeply anesthetized at 19:00 ZT (5 h before artificial sunset) and transcardially perfused with phosphate-buffered saline (PBS) followed by 10% neutral-buffered formalin. The brains were then post-fixed in formalin, embedded in paraffin, and sectioned coronally at a thickness of 4 μm. Sections were taken at −2.055 mm (minus 2.055 mm) from the bregma according to the Allen Brain Institute’s mouse brain atlas (atlas.brain-map.org accessed on 1 January 2025).

Before staining, sections were deparaffinized in xylene and rehydrated through a graded ethanol series. Antigen retrieval was performed by incubating slides in citrate buffer (pH 6.0, 0.05% Tween-20) at 97 °C for 30 min in a water bath. Sections were then permeabilized with 1% Triton X-100 in PBS for 30 min at room temperature and blocked in 5% BSA with 1% Triton X-100 in PBS for 1 h. Sections were then incubated overnight at 4 °C in a humidified chamber with primary antibodies against post-translational histone modifications (Table 1). After three washes in PBS, sections were incubated with secondary antibodies (Table 1) for 2 h at room temperature. Following three additional PBS washes, nuclei were counterstained with DAPI for 5 min. Finally, slides were mounted with Shandon Immu-Mount (Thermo Scientific, Waltham, MA, USA #9990402) and imaged on an upright epifluorescence microscope (Axio Imager M2 Colibri 7, Carl Zeiss, Jena, Germany) using a 40× objective. For each animal, ten non-overlapping fields of view were analyzed from both the left and the right somatosensory cortex (20 fields total). Imaging parameters were kept constant across all samples. For representative examples of stainings, see Figure S5.

Cell nuclei were automatically detected by thresholding the DAPI channel, with the threshold set to the local mean intensity (61 × 61 pixel area). We refined the shapes of the nuclear contours by applying five iterations of morphological opening to the binary image. The resulting nuclear contours are displayed on the DAPI channel in Figure S5. Histone modification was quantified as a mean intensity for the respective channel, normalized by DAPI channel intensity.

### 4.6. Statistics

Analysis of covariance (ANCOVA) was used to compare animal body masses (Figure 1) at different ages, treating groups (CNT, 35 °C, 37 °C) as a categorical factor and number of littermates as a continuous factor. A post hoc Fisher’s LSD test was applied to determine the statistical significance of inter-group differences. For the remaining statistical comparisons, the Mann–Whitney U-test was used. Classical cosinor regression was used to determine the acrophases and amplitudes of the behavioral time series. The data are presented as the mean value ± standard error of the mean (M ± SEM). In the boxplots, the box extends from the first quartile (Q1) to the third quartile (Q3) of the data, with a line at the median and a rhombus at the mean. The boxplot whiskers extend from the box to the farthest data point lying within 1.5 times the inter-quartile range (IQR = Q3 − Q1) from the box. All analyses were performed using Python.

## Figures and Tables

**Table 1:** Primary and Secondary Antibodies Used.

Catalog No.	Manufacturer	Dilution	Host Species	Target
05-1951	Millipore (Waltham, MA, USA)	1:500	Mouse	H3K27me3
4909	Cell Signaling (Waltham, MA, USA)	1:200	Rabbit	H3K36me3
ab1220	Abcam (Fremont, CA, USA)	1:200	Mouse	H3K9me2
ab150077	Abcam (Fremont, CA, USA)	1:600	Goat	Alexa Fluor 488 anti-rabbit IgG
2253917	Invitrogen (Carlsbad, CA, USA)	1:250	Goat	Alexa Fluor 555 anti-mouse IgG

## Data Availability

The data and code that support the findings of this study are available at https://doi.org/10.6084/m9.figshare.30073087 accessed on 8 September 2025.

## Supplementary Materials

The following supporting information can be downloaded at: https://www.mdpi.com/article/10.3390/ijms262110360/s1.

## Abbreviations

ART	Assisted Reproductive Technologies
COCs	Cumulus-Oocyte Complexes
IVF	In Vitro Fertilization
H3K9me2	Di-methylation at the 9th lysine residue of the histone H3 protein
H3K27me3	Tri-methylation at the 27th lysine residue of the histone H3 protein
H3K36me3	Tri-methylation at the 36th lysine residue of the histone H3 protein
RER	Respiratory Exchange Ratio (the ratio of VCO_2_ to VO_2_)
VCO_2_	Volume carbon dioxide production
VO_2_	Volume oxygen consumption
ZGA	Zygotic Genome Activation
Statistical abbreviations	
ANCOVA	Analysis of covariance
AUC	Area Under the Curve
LSD	Fisher’s Least Significant Difference test
