# Supplementary material for: IVF and Thermal Manipulation at the First Cleavage Stage Alter Offspring Circadian Phenotype, Sleep, and Brain Epigenetics
Source: Int J Mol Sci. 2025 Oct 24;26(21):10360. doi: 10.3390/ijms262110360 (PMC12607329; doi:10.3390/ijms262110360)
Supplement: Supplementary file 1 [file ijms-26-10360-s001.zip › ijms-3886544-supplementary.pdf]

Supplemental materials for IVF and Thermal Manipulation at the First Cleavage Stage Alter Offspring Circadian Phenotype, Sleep, and Brain Epigenetics.

Daniil Zuev [1], Aliya Stanova [1], Galina Kontsevaya [1], Alexander Romashchenko [1,2], Nikita Khotskin [1], Marina Sharapova [1], Mikhail Moshkin [1,3], Ludmila Gerlinskaya [1*], Yuri Moshkin [1,4*]

1. Federal Research Center Institute of Cytology and Genetics, Siberian Branch of RAS, Russia, 630090 Novosibirsk; zuevdaniil.zuevdaniil@gmail.com (D.Z.); aliya.stanova@mail.ru (A.S.); koncevayagalina@bionet.nsc.ru (G.K.); arom2006@gmail.com (A.R.); khotskin@bionet.nsc.ru (N.K.); barberry1505@gmail.com (M.S); mmp@bionet.nsc.ru (M.M.); lgerlinskaya@gmail.com (L.G); yury.moshkin@gmail.com (Y. M.).

2. LIFT Center LLC, 121205 Moscow, Russia

3. Tomsk State University, Institute of Biology, Ecology, Soil Science, Agriculture and Forestry; Department of Vertebrate Zoology and Ecology, 634050 Tomsk, Russia.

4. Gene Learning Association, 1205 Geneva, Switzerland

* Correspondence: lgerlinskaya@gmail.com, lgerlinskaya@bionet.nsc.ru, yury.moshkin@gmail.com

S1

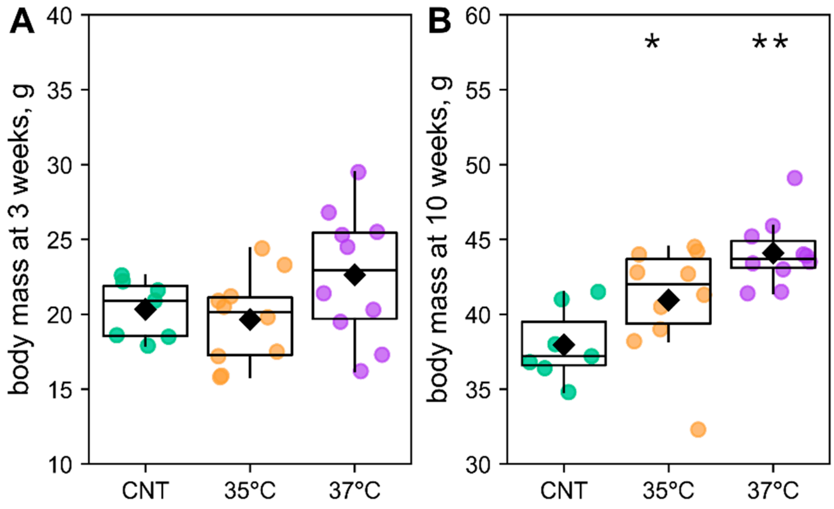

Figure S1. *Body mass of male offspring selected for further experiments at (A) 3 weeks and (B) 10 weeks of age; * p < 0.05, ** p < 0.01 Mann-Whitey U-test IVF groups vs control.*

S2

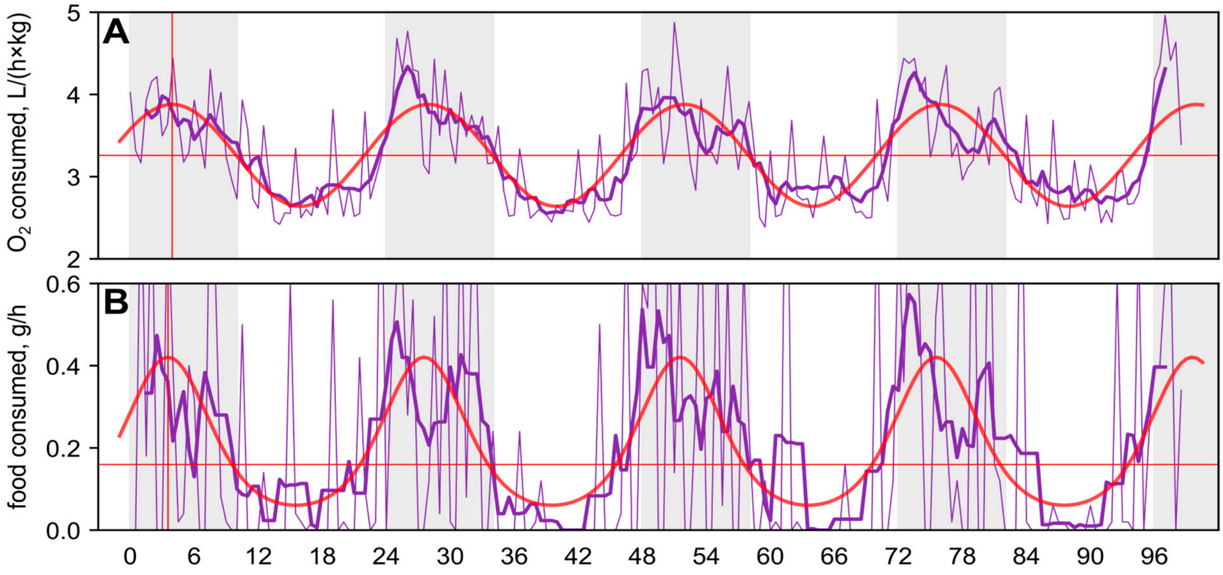

Figure S2 *Example fits of the Cosinor model. (A, B) Sample model fits for (A) oxygen consumption and (B) food consumption in a single animal. Measured values (thin magenta line) and their sliding window average (thick magenta line) are shown alongside the cosinor prediction (red line). Acrophase (red vertical line), mesor (horizontal line), and dark phase (gray shading) are indicated.*

S3

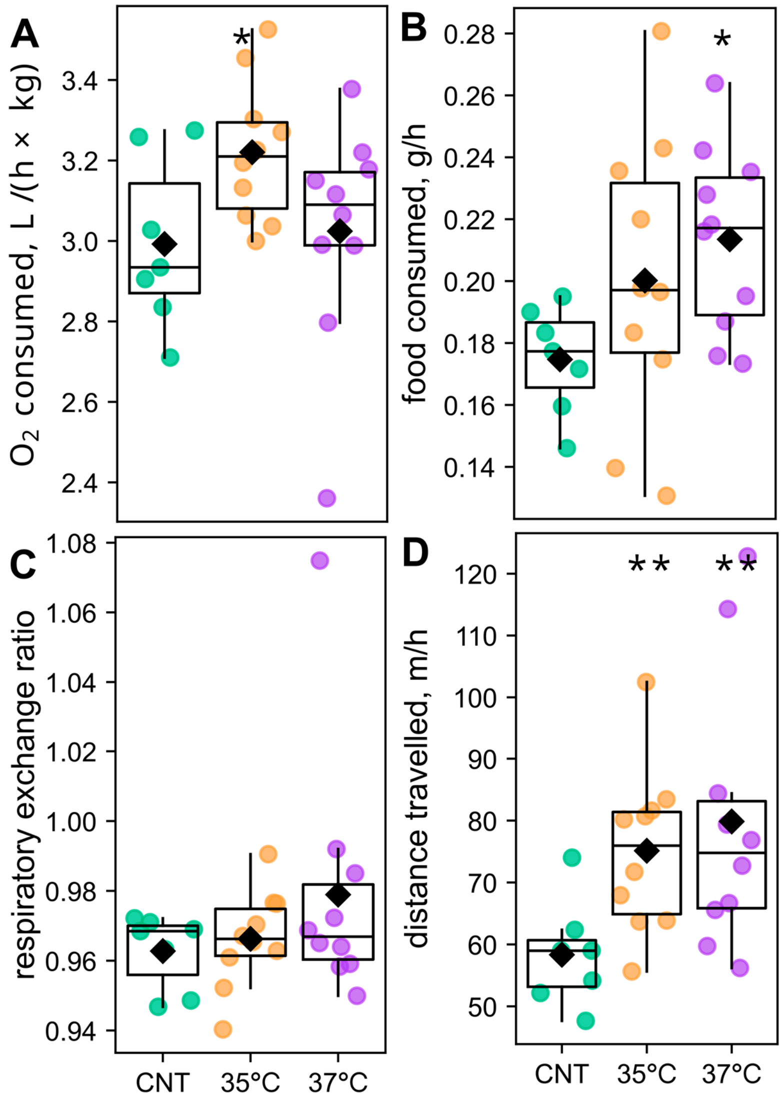

Figure S3. *Cosinor analysis of circadian metabolic and behavioral parameters. Mesor of (A) oxygen consumption (L/h/kg), (B) food intake (g/h), (C) respiratory exchange ratio (RER), and (D) distance traveled (m/h). *p < 0.05, **p < 0.01 (Mann-Whitney U-test).*

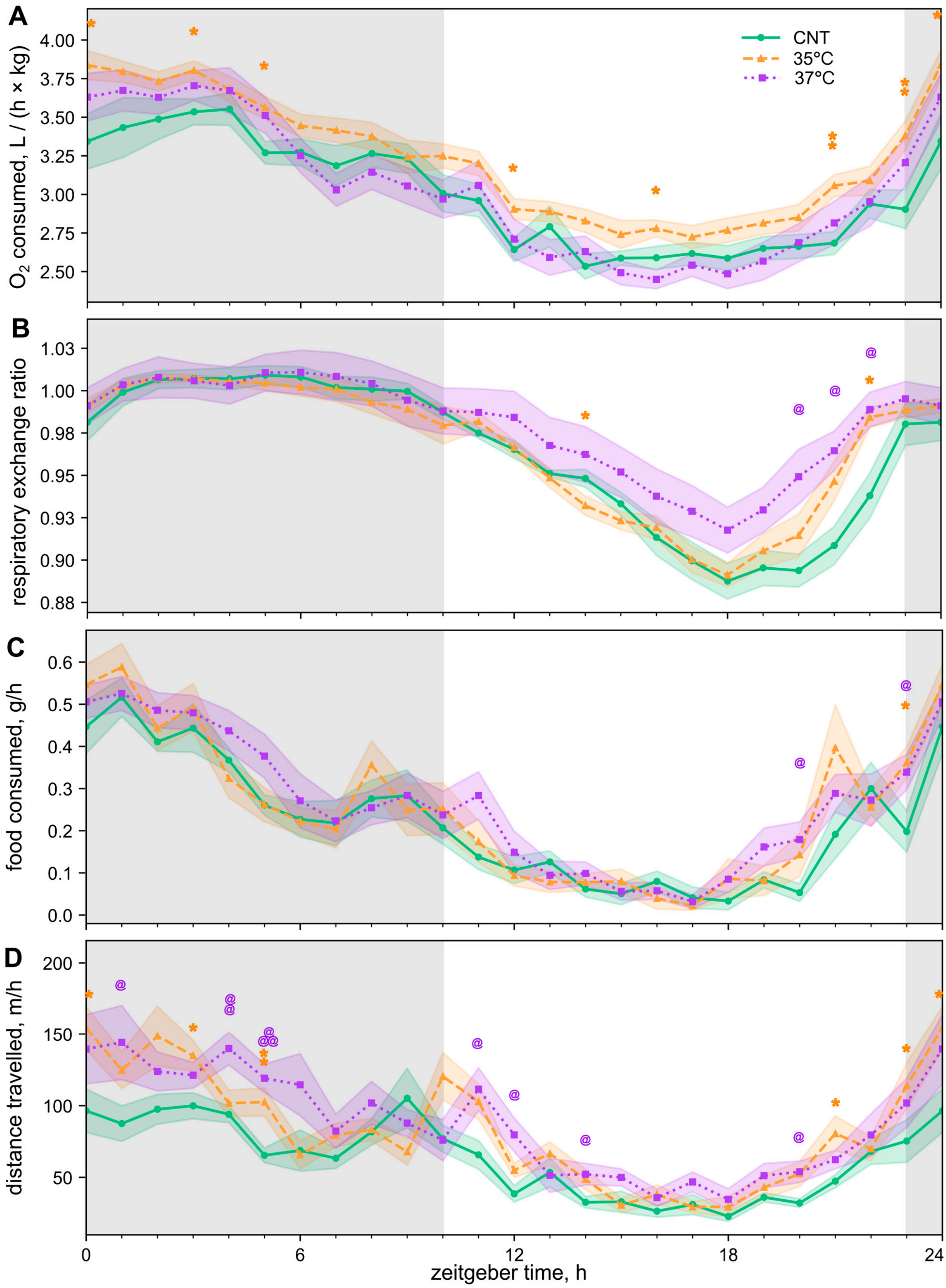

Figure S4. *Circadian profiles of (A) oxygen consumption, (B) respiratory exchange ratio (RER), (C) food intake, and (D) locomotor activity (distance traveled). Data are presented as mean (lines) and the standard error of the mean (ribbons). The shaded gray area represents the lights-off (dark) period. Significance (Mann-Whitney U-test) of 35°C vs. control: * - p < 0.05, ** - p < 0.01, *** - p < 0.001; and of 37°C vs. control: @ - p < 0.05, @@ - p < 0.01, @@@ - p < 0.001.*

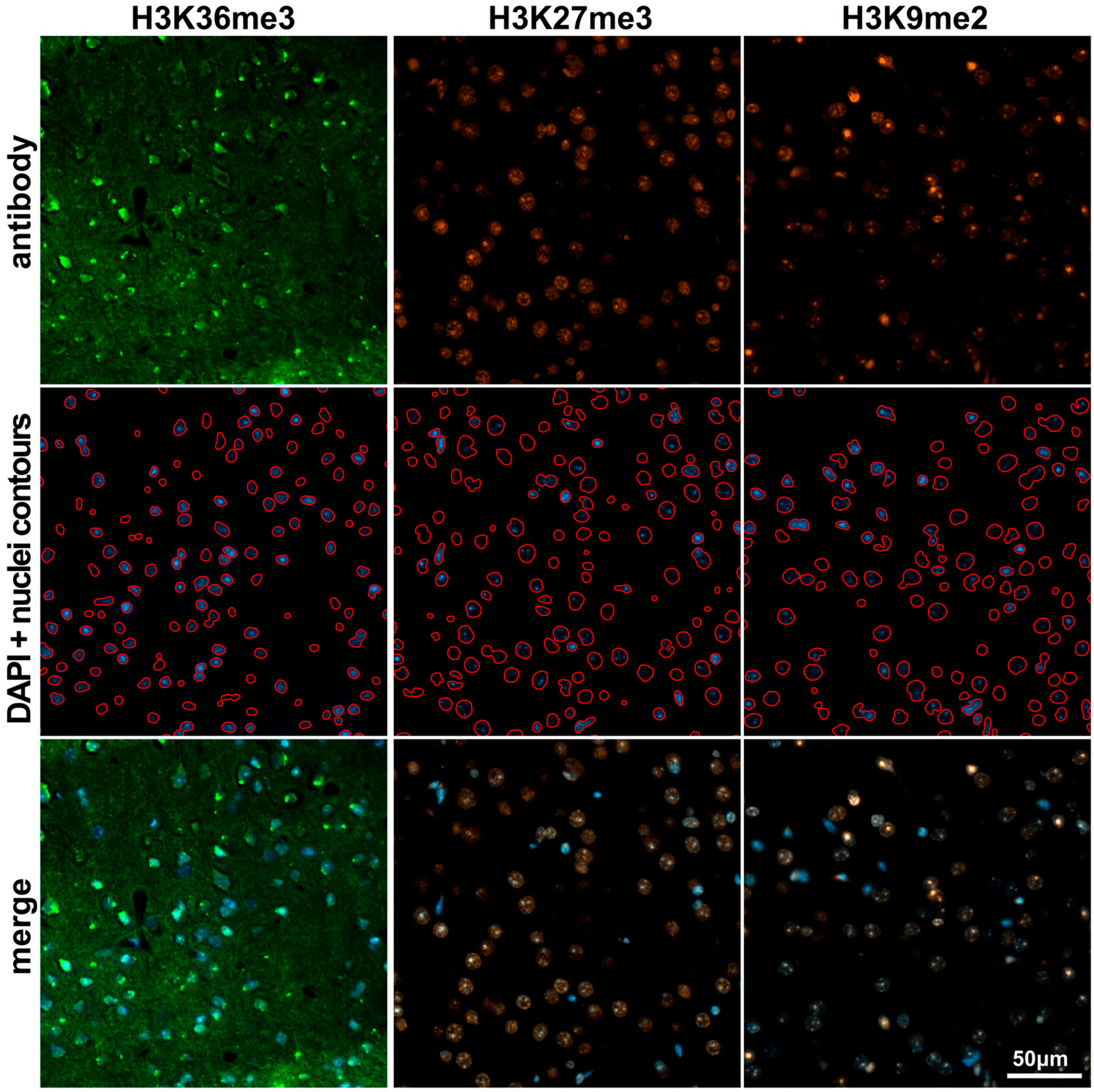

Figure S5. *Representative fluorescent images of the mouse somatosensory cortex immunostained for histone modifications. Nuclei are counterstained with DAPI (blue), and automated cell segmentation boundaries are outlined in red.*
